# Unusual Foreign Body of Parotid Gland Presenting as Sialolithiasis: Case Report and Literature Review

**DOI:** 10.1155/2012/367349

**Published:** 2012-12-23

**Authors:** Sivapatha Sundaram Sreetharan, Rajan Philip

**Affiliations:** ^1^Department of Otorhinolaryngology, Head and Neck Surgery, Hospital Pantai Batu Pahat, 9S, Jalan Bintang Satu, Taman Koperasi Bahagia, 83000 Batu Pahat, Johor, Malaysia; ^2^Department of Otorhinolaryngology, Head and Neck Surgery, Hospital Raja Permaisuri Bainun, Ipoh, Jalan Hospital, 30990 Ipoh, Perak, Malaysia

## Abstract

This case report highlights an unusual case where a foreign body in the parotid gland was initially thought to be sialolithiasis based on CT scans. The foreign body was safely retrieved from the parotid gland without formal superficial parotidectomy using methylene blue and an image intensifier to localize the lesion. Diagnosis and management of foreign bodies in the parotid gland are reviewed, and surgical options in removal of such lesions are discussed.

## 1. Introduction

Patients presenting with pain and swelling over the parotid gland usually have infective or obstructive sialadenitis. Obstructive sialadenitis may be due to calculi, fibromucinous plugs, duct stenosis, foreign bodies, anatomic variations, or malformations of the duct system leading to a mechanical obstruction associated with stasis [1]. Sialolithiasis accounts for 66% of obstructive salivary disease [2]. However, only 5%–10% of cases involve the parotid gland, the majority being in the submandibular gland [3].

Strictures and kinks are the second most frequent cause of obstructive sialadenitis and involve the parotid duct in 75.3% of cases [4]. Foreign bodies causing obstructive parotid sialadenitis are extremely rare. Only a handful of cases have been reported of foreign bodies in the parotid gland, and most were penetrative foreign bodies from the skin. This case demonstrates the diagnostic difficulties and management options available in the management of this condition.

## 2. Case Report

A 63-year-old lady complained of left facial pain for 10 days. She has had a left facial swelling for 3 years which was not increasing in size. The pain was not exacerbated after meals, and she had no recollection of ingestion of any foreign bodies. She previously had a CT scan performed for the same condition. She was informed that she had calculi in the left parotid gland and was advised of surgery to remove it.

On clinical examination, the left parotid gland was slightly enlarged but was not inflamed. On examination of the oral cavity, no pus was seen form the left parotid duct. No neck nodes were palpable, and the right parotid was normal.

**Figure 1:**
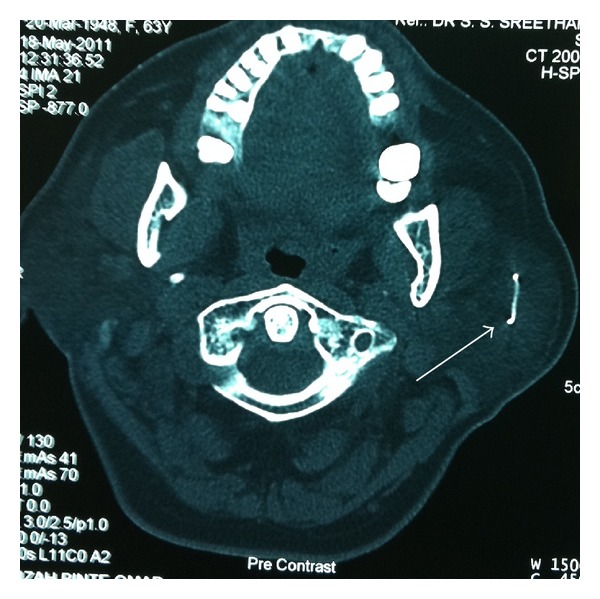
CT scan showing a linear calcification in the substance of the parotid gland (arrow).

CT scan showed enlarged and diffusely enhanced left parotid gland with a 17 mm linear calcification (Figure 1). There was no evidence of any abscess. Bilateral periparotid and level 2 and 3 neck shotty lymph nodes were noted. A diagnosis of left parotid sialolithiasis was made, and the patient was planned for exploration of the parotid gland and removal of the calculi.

**Figure 2:**
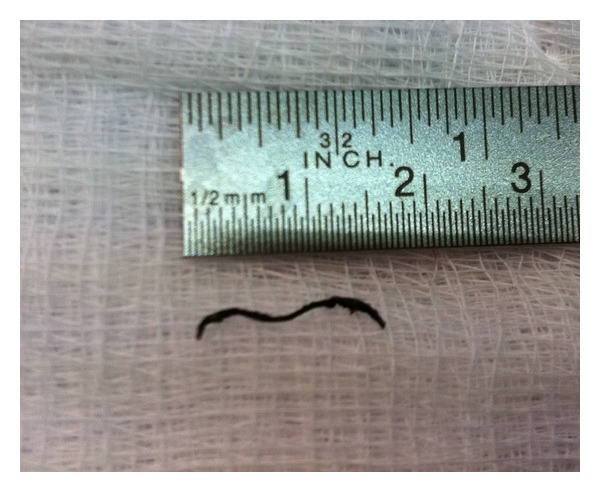
The foreign body which was a stapler wire.

On the operation table, the calculi were localized using an image intensifier. Methylene blue dye was infiltrated right down into the estimated location of the lesion. A mini face-lift incision was made, and the skin flap was elevated. After elevation of the superficial musculoaponeurotic system (SMAS), the parotid gland was carefully dissected following the track marked out by the methylene blue. A 1.7 cm metallic wire was found and removed (Figure 2). There were no calculi or pus noted. The rest of the gland appeared normal. The wound was closed without any drain. The patient recovered well, and there was no facial nerve palsy.

## 3. Discussion

Foreign bodies of the parotid glands are fairly infrequent. A search of Medline using the key words foreign body and parotid gland revealed 19 of such cases. The first case was published way back in 1958 [5]. In most of the cases the foreign body was penetrative from the skin. Among the foreign body reported were pieces of glass, pencil nib, wood fragments, metallic foreign body, and a piece of hair. In this case it is uncertain how the stapler wire ended up in the parotid gland as there was no history of preceding facial trauma. The most likely explanation is via accidental ingestion and retrograde migration through Stensen's duct [6].

This patient presented with a parotid swelling for 3 years and recurrent episodes of pain. The differential diagnosis would be sialadenitis, sialolithiasis, ductal anomalies, mucus plugs, and tumor compression. In this patient the CT scan was used as the first line imaging modality as it is able to distinguish between the differentials and aid in planning the surgical approach. Standard radiography is seldom employed as it can only detect radioopaque bodies and is not helpful in cases of abscess and tumors. Even in cases of calculi, it is unable to reveal radiolucent, intraglandular, and small stone in 20% of cases [7]. However in this case the CT scan was misleading because the foreign body had elicited an inflammatory reaction which appeared as a linear calcification.

In experienced hands, ultrasonography may be a good first line investigative modality. Sialography is often performed to outline the ductal architecture and level of obstruction. Newer investigative modalities include magnetic resonance (MR) sialography which has the advantage of not needing contrast injection and ductal cannulation and allows for functional evaluation of the gland. Sialendoscopy has emerged as an excellent diagnostic and therapeutic instrument which allows excellent visualization of the ductal tracts [8].

Removal of the foreign body presents a challenge. Traditionally, formal parotid incision and identification of the facial nerve would have been undertaken before removal of the foreign bodies. Because of the morbidity associated with this method, less invasive and gland sparing methods are now preferred [9]. Sialendoscopy has a well-recognized role in removal of mobile parotid calculi but there has been no report of it being used in foreign body retrieval [1].

In this case the foreign body was too proximal and deeply embedded to have been removed solely with sialoendoscopes. However combined sialoendoscopy and open method which has successfully been used for large proximal calculi may have been possible. In that approach a flexible light source is introduced with the sialoendoscope until the level of obstruction. Careful dissection of the parotid gland under magnification with facial nerve monitoring is undertaken until the calculi are located [10]. In this case, the image intensifier was used to localize the stone, and the pathway for dissection was marked out by methylene blue infiltration into the parotid gland. Careful dissection and the use of the facial nerve stimulator enabled retrieval of the foreign body.

## 4. Conclusion

Recurrent parotitis due to Stensen's duct obstruction is commonly caused by strictures or calculi. It is rarely caused by foreign bodies. This case demonstrates that CT scans can misdiagnose foreign bodies in the parotid gland and that in selected cases a less invasive procedure can be successfully performed with the concurrent use of imaging and neuromonitoring.
